# Receiving Post-Conflict Affiliation from the Enemy's Friend Reconciles Former Opponents

**DOI:** 10.1371/journal.pone.0013995

**Published:** 2010-11-15

**Authors:** Roman M. Wittig, Christophe Boesch

**Affiliations:** 1 Max Planck Institute for Evolutionary Anthropology, Leipzig, Germany; 2 School of Psychology, University of St. Andrews, St. Andrews, United Kingdom; Università di Parma, Italy

## Abstract

The adaptive function of bystander initiated post-conflict affiliation (also: consolation & appeasement) has been debated for 30 years. Three influential hypotheses compete for the most likely explanation but have not previously been tested with a single data set. The consolation hypothesis argues that bystander affiliation calms the victim and reduces their stress levels. The self-protection hypothesis proposes that a bystander offers affiliation to either opponent to protect himself from redirected aggression by this individual. The relationship-repair hypothesis suggests a bystander can substitute for a friend to reconcile the friend with the friend's former opponent. Here, we contrast all three hypotheses and tested their predictions with data on wild chimpanzees (*Pan troglodytes verus*) of the Taï National Park, Côte d'Ivoire. We examined the first and second post-conflict interactions with respect to both the dyadic and triadic relationships between the bystander and the two opponents. Results showed that female bystanders offered affiliation to their aggressor friends and the victims of their friends, while male bystanders offered affiliation to their victim friends and the aggressors of their friends. For both sexes, bystander affiliation resulted in a subsequent interaction pattern that is expected for direct reconciliation. Bystander affiliation offered to the opponent's friend was more likely to lead to affiliation among opponents in their subsequent interaction. Also, tolerance levels among former opponents were reset to normal levels. In conclusion, this study provides strong evidence for the relationship-repair hypothesis, moderate evidence for the consolation hypothesis and no evidence for the self-protection hypothesis. Furthermore, that bystanders can repair a relationship on behalf of their friend indicates that recipient chimpanzees are aware of the relationships between others, even when they are not kin. This presents a mechanism through which chimpanzees may gain benefits from social knowledge.

## Introduction

A fundamental question in Behavioural Ecology is the evolution of sociality. Living in social groups provides several benefits such as better defense of food resources, less predation pressure and increased benefits from pooling information [1], [2]. Increased sociality seems to enhance the reproductive success in a wide range of group living animals, from primates to birds (*Papio spec.*: [3], [4]; *Equus equus*: [5]; *Uria aalge*: [6]). Social animals, however, also have to cope with a variety of costs incurred through group living, such as competition from group members, risk of infanticide and increased threat of disease transmission [1], [2]. These tradeoffs favor the evolution of behavioral strategies that enable individuals to increase the benefits that they gain and minimize the costs that they incur by living in social groups.

Competition among group members often escalates into aggression, which disrupts sociality. Sometimes the disruptive effects of aggression appear to be mitigated by post-conflict friendly behavior between recent opponents [7]. Such reconciliation appears to reduce stress induced by aggression [8], [9] and restores opponents' mutual tolerance to baseline levels [10], [11]. Reconciliation attempts, however, can be blocked when victims of aggression avoid recent opponents in the apparent fear that aggression will be renewed. In some cases, upon observing the fight, an uninvolved third party (called *bystander*) may offer an affiliative behavior to either the aggressor or victim of the fight (called *affiliation recipient*). Like with reconciliation, such post-conflict affiliation with a bystander seems able to reduce aggression-induced stress [12].

Since its first description by de Waal & van Roosmalen [13], post-conflict affiliation initiated by a bystander has been labeled ‘consolation’. During the ongoing debate over its function, this post-conflict affiliation was relabeled as ‘consolation’ only when given to the victim of aggression, and ‘appeasement’, when given to the aggressor [14]. Both labels, however, are implying a function, although the act of ‘consolation’ and ‘appeasement’ may contain several functions [15]. Therefore we are using the descriptive term ‘post-conflict affiliation initiated by a bystander’ with the victim or with the aggressor, respectively – or for short *bystander affiliation*.

Three main hypotheses shape our understanding of the function and the cognitive underpinnings of bystander affiliation [15]. The *consolation* hypothesis predicts that *bystander affiliation* alleviates the recipient's stress caused by the conflict [12]–[14]. In this case, the bystander's motivation for offering affiliation is deemed to be empathetic and should increase with bond strength between the bystander and the affiliation recipient. Thus, bystander affiliation should be initiated primarily by an individual closely bonded to the affiliation recipient, while the strength of the bystander's bond to the recipient's opponent is irrelevant.

Secondly, the *self-protection* hypothesis anticipates that the bystander offers affiliation to the recipient in order to avoid becoming the target of redirected aggression [16], [17]. Redirected aggression, or attacking a bystander, can be used by either opponent of the conflict to reduce stress levels and deflect aggressive attention [18]. Here, the bystander's motivation to offer affiliation should be determined by the bystander's relationship with the affiliation recipient. Motivation should increase with decreasing bond strength, since close bonding partners are unlikely to face redirected aggression [16].

Finally, the *relationship-repair* hypothesis predicts that a bystander offering affiliation repairs the opponents' relationship and reduces the aggression-induced stress [19]–[21]. This may happen if the bystander is a close bonding partner of the affiliation recipient's opponent. Affiliating with the affiliation recipient results in reconciliation on the opponent's behalf. Under this condition the bystander should be a close bonding partner to the recipient's opponent, but the relationship with the affiliation partner is irrelevant [15], [20].

Relationship quality has been identified as the key to understanding the different functions of bystander affiliation [15]. Here, we investigated all three hypotheses using data from a community of wild chimpanzees in the Taï National Park, Côte d'Ivoire. We analyzed bystander affiliation according to the *relationship benefit index* (RBI: Table 1) the bystander had with either the aggressor or the victim of aggression (the RBI ranges between 3 and 1 with RBI = 3 indicating a good friend, RBI = 2 reflecting a weak friend and a RBI = 1 representing non-friend). The relationship benefit index has been shown to determine the rate of reconciliation [11], [22] and to best reflect the relationship quality of Taï chimpanzees [21].

**Table 1:** Relationship benefit index (RBI) of all subject – subject dyads in the North group of Taï chimpanzees.

	♂				♀											
	BRU	MAC	MAR	NIN	BEL	CAS	DIL	FOS	GOM	LOU	MYS	NAR	PER	RIC	VEN	mean RBI
BRU																1,857
MAC	2															2,214
MAR	3	2														1,857
NIN	1	1	3													1,857
BEL	2	3	3	2												1,786
CAS	3	2	1	3	2											2,214
DIL	1	2	1	2	3	2										1,857
FOS	2	3	1	2	1	3	1									1,571
GOM	2	2	2	1	1	2	2	1								1,5
LOU	2	3	2	2	2	2	2	1	1							1,929
MYS	1	3	2	2	1	3	3	2	3	2						2,143
NAR	1	1	2	2	1	2	2	2	1	2	3					1,643
PER	2	2	1	1	1	2	2	1	1	2	2	2				1,571
RIC	1	2	1	3	2	2	1	1	1	1	2	1	2			1,5
VEN	3	3	2	1	1	2	2	1	1	3	1	1	1	1		1,643

The RBI is a composite index from two variables: food sharing and agonistic support (see methods). RBI: (1) low benefit partner  =  non friends, (2) medium benefit partner  =  weak friends, (3) high benefit partner  =  good friends.

**Figure 1:**
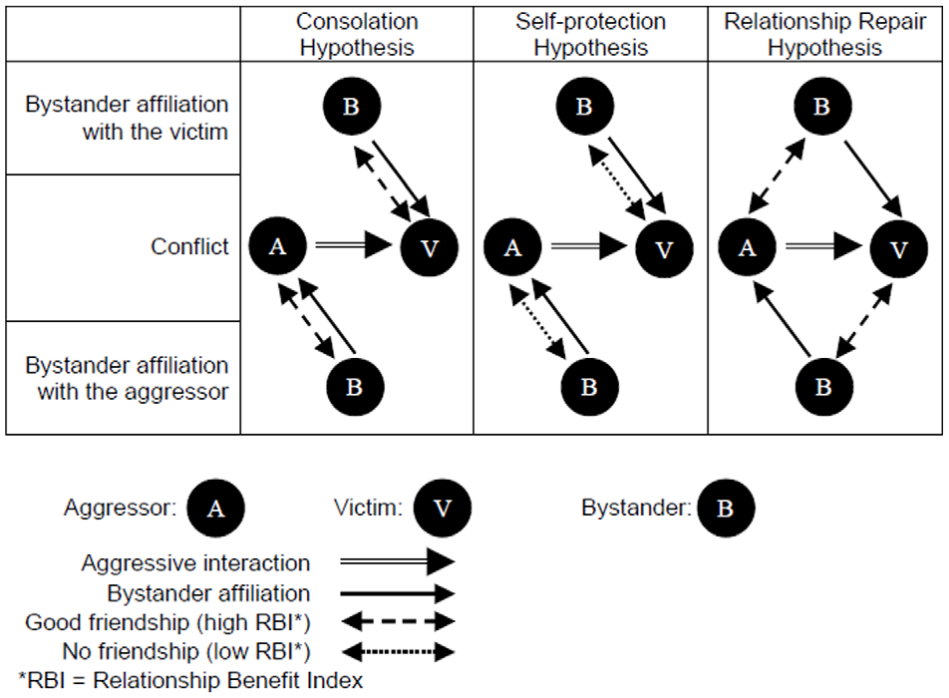
Predicted relationships between the bystander (B) and both of the opponents (A: aggressor, V: victim) for each of the three hypotheses are shown separately for bystander affiliation with the victim (top) and with the aggressor (bottom). Solid lines show the observed interactions (conflict: double line; bystander affiliation: single line), discontinuous lines show the predicted relationship (dashed: good relationship; dotted: bad relationship), while no line indicates no predicted relationship.

To test the three hypotheses we investigated the first and second post-conflict interactions with respect to both the dyadic and triadic relationships between the bystander and the two opponents. Each of the hypotheses predicts a different pattern of the bystander's relationship with both the affiliation recipient and the recipient's opponent. This allows us to test the predictions of all three hypotheses at once. We examined how the bystander's relationships with both the affiliation recipient and the recipient's opponent affected the first and second post-conflict interactions. We tested the following predictions with respect to the first (Figure 1) and second post-conflict interactions (predictions regarding the second post-conflict interaction were tested only when the predictions for the first post-conflict interaction were met):

(1) *Consolation* hypothesis: Friends are expected to react more empathetically to each others' distress than non-friends [23] and close bonding partners are better in reducing stress levels than weak bonding partners [24], [25]. Therefore, consolation is most likely when a bystander offers affiliation to a friend. This hypothesis requires a dyadic relationship assessment by the affiliation recipient. Following the *consolation* hypothesis, in the first post-conflict interaction, we predict that the bystander's RBI with the affiliation recipient is higher than the bystander's average RBI (where the ‘bystander’s average RBI' was calculated as the mean RBI of all dyads for this particular bystander). If consolation has occurred we predict that individuals will have been calmed. To measure this we examine the second post-conflict interaction: we predict that chimpanzees are more likely to engage in friendly, as opposed to aggressive, interactions with others after being offered bystander affiliation by a friend with a high RBI.

(2) *Self-protection* hypothesis: A bystander risks being the target of redirected aggression, especially from a ‘non-friend’ victim [15], [16]. Similar to the *consolation hypothesis*, the strength of the friendship between the bystander and the affiliation recipient determines the likelihood of self-protection. Therefore, following the *self-protection* hypothesis, we predict that during the first post-conflict interaction the bystander's RBI with the affiliation recipient is lower than the bystander's average RBI. The second post-conflict interaction determines if the self-protection function is effective: we predict that non-friend bystanders receive less aggression by the recipient after initiating bystander affiliation.

(3) *Relationship-repair* hypothesis: Only a close bonding partner of the recipient's opponent seems able to effectively repair the relationship between the two opponents [20], [21]. This implies a triadic relationship assessment by the recipient of affiliation in order for the affiliation recipient to assess the relationship between the bystander and the former opponent to determine if reconciliation may have taken place. Following the *relationship-repair* hypothesis, in the first post-conflict interaction, we predict that the bystander's RBI with the recipient's opponent is higher than the bystander's average RBI. The second post-conflict interaction determines if the relationship-repair among the former opponents was effective: we predict that former opponents are more likely to engage in friendly interactions when the bystander's RBI with the recipient's opponent is high. In addition, we predict that tolerance among opponents (measured by the latency of the friendly second post-conflict interaction among the opponents) returns to baseline levels, only if a good friend of the recipient's opponent affiliated with the affiliation recipient.

Previous analysis of the data has shown that bystander initiated post-conflict affiliation in Taï chimpanzees is a true post-conflict interaction [22]. Since sex differences are apparent in the pattern of the Taï chimpanzee's post-conflict management [22], we differentiated in our analysis between male and female bystanders as well as between affiliations with the victim (‘consolation’) and with the aggressor (‘appeasement’).

## Results

We observed at total 876 aggressive interactions (see [22]). A bystander immediately offered affiliation to one of the opponents in 164 cases (18.7% of aggressive interactions). The observed frequency very likely underestimated the actual frequency by 50%, as we followed only one opponent out of two. The bystander was an adult in 66.5% of the observed cases of bystander affiliation (N = 109). In 26 events bystanders affiliated with the victim and in 83 events they affiliated with the aggressor.

### The effect of the bystander's relationships on bystander affiliation with the victim during the first post-conflict interaction (also called ‘consolation’)

Twelve adult bystanders of either sex offered affiliation to the victim (Table 2). For each bystander, we calculated their average relationship benefit index (RBI) with all individuals. The mean RBI across all twelve bystanders was RBI_MF_±SD = 1.85±0.24. The median RBI for males (RBI_M_ = 1.86) was similar to that for females (RBI_F_ = 1.79; average difference = 0.07, CI_LOW_ = −0.07, CI_HIGH_ = 0.57, N_M_ = 3, N_F_ = 9).

**Table 2:** Bystander affiliation with the victim: Mean relationship benefit index (RBI) of bystanders (N = 12) with the aggressor and with the victim and the percentage of friendly interactions following bystander affiliation.

					opponents	victim and any group member
bystander	sex	N	mean RBI with aggressor (opponent)	mean RBI with victim (affiliation recipient)	exchange a friendly interaction after bystander affiliation with the victim	
MAC	♂	2	1.5	3	50%	100%
MAR	♂	1	2	2	100%	100%
NIN	♂	1	1	2	0%	-*
BEL	♀	3	3	2.67	67%	67%
CAS	♀	3	2	1.67	67%	67%
DIL	♀	3	2	1.33	33%	33%
FOS	♀	2	3	1	100%	100%
GOM	♀	2	2	2	50%	50%
LOU	♀	2	2.5	2	50%	100%*
MYS	♀	4	2.75	2	50%	50%
NAR	♀	1	1	1	0%	0%
VEN	♀	2	3	2	50%	50%
mean	all	12	2.15	1.89		
mean	♂	3	1.5	2.33		
mean	♀	9	2.36	1.74		

*one sample excluded, since victims did not interact with anybody for the rest of the observation time following bystander affiliation.

We subtracted the bystander's average RBI from the bystander's specific RBI with that affiliation recipient (here the victim of aggression) and likewise from the bystander's RBI with the opponent (here the aggressor). For both samples (of subtraction terms), we calculated the 5% confidence limits on the average of the sample using bootstrap methods.

We found neither differences between the bystanders' average RBIs and the bystanders' RBIs with the affiliation recipients (mean = 0.04, CI_L_ = −0.21, CI_H_ = 0.35, N = 12) nor with the recipient's opponents (mean = 0.30, CI_L_ = −0.11, CI_H_ = 0.68, N = 12) when pooling males and females. When analyzed separately, however, female bystanders proved to have significantly higher RBIs than average with the opponent's recipients (mean = 0.55, CI_L_ = 0.07, CI_H_ = 0.98, N = 9) whilst their RBIs with the affiliation recipients were indifferent from the average RBIs (mean = −0.07, CI_L_ = −0.36, CI_H_ = 0.34, N = 9). All three males, in contrast, had higher RBIs than average with the affiliation recipients (median = 0.14, range = [0.14,0.79], N = 3) whilst the males' RBIs with the recipient's opponents were sometimes above and sometimes below average (median = −0.71, range = [−0.86, 0.14], N = 3).

In sum, female bystanders were more likely to affiliate with the victim of aggression, if they had a good friendship with the opponent. Male bystanders seemed more likely to affiliate with victims who were their good friends.

### The effect of the bystander's relationships on bystander affiliation with the aggressor during the first post-conflict affiliation (also called ‘appeasement’)

All potential fifteen adult bystanders of both sexes initiated bystander affiliation with the aggressor (Table 3). The average relationship benefit index (RBI) across all 15 bystanders was RBI_MF_±SD = 1.81±0.24. The median RBI for males (RBI_M_ = 1.86) was similar to that for females (RBI_F_ = 1.64; average difference = 0.22, CI_L_ = 0.00, CI_H_ = 0.47, N_M_ = 4, N_F_ = 11).

**Table 3:** Bystander affiliation with the aggressor: Mean relationship benefit index (RBI) of bystanders (N = 15) with the aggressor and with the victim and the percentage of friendly interactions following bystander affiliation.

					opponents	aggressor and any group member
bystander	sex	N	mean RBI with aggressor (affiliation recipient)	mean RBI with victim (opponent)	exchange a friendly interaction after bystander affiliation with the aggressor	
BRU	♂	4	1.75	2.5	25%	33%*
MAC	♂	5	2.6	2.4	20%	50%*
MAR	♂	8	2	3	63%	83%*
NIN	♂	7	1.29	2.57	71%	100%*
BEL	♀	5	3	1.8	20%	20%
CAS	♀	7	1.71	1.86	57%	67%*
DIL	♀	2	1.5	2	50%	50%
FOS	♀	5	2	2	80%	80%
GOM	♀	6	2	1.67	67%	67%
LOU	♀	4	2.75	2.25	75%	75%
MYS	♀	7	3	2	43%	57%
NAR	♀	3	1.67	1.67	33%	100%
PER	♀	7	2	1.14	29%	29%
RIC	♀	8	1.88	2.25	38%	38%
VEN	♀	5	3	1.6	40%	67%*
mean	all	15	2.14	2.05		
mean	♂	4	1.91	2.62		
mean	♀	11	2.23	1.84		

*samples excluded, since aggressors did not interact with anybody for the rest of the observation time following bystander affiliation.

As above, we calculated the two differences between the bystander's average RBIs and the bystander's RBIs with the affiliation recipients (here the aggressor) and the recipient's opponents (here the victim of aggression). Afterwards we conducted the bootstrap sampling again.

We found that bystanders had significantly higher RBIs than average with both opponents, the affiliation recipients (mean = 0.33, CI_L_ = 0.07, CI_H_ = 0.59, N = 15) and the recipient's opponents (mean = 0.24, CI_L_ = 0.06, CI_H_ = 0.45, N = 15), when pooling males and females. When analyzing the sexes separately, female bystanders proved to have significantly higher RBIs than average with their affiliation recipients (mean = 0.47, CI_L_ = 0.13, CI_H_ = 0.78, N = 11), whilst their RBIs with the recipient's opponents was indifferent to their average RBI (mean = 0.08, CI_L_ = −0.10, CI_H_ = 0.29, N = 11). In contrast, all four males had higher RBIs than average with the recipient's opponents (median difference = 0.68, range = [0.19,1.14], N = 4), whilst the males' RBIs with the recipient were above and below average (median difference = 0.02, range = [−0.57,0.39], N = 4).

Thus, bystanders were more likely to affiliate with the aggressor when they had good friendships with both the affiliation recipients and the recipient's opponents. Females were more likely to affiliate with their friend aggressor. Males, in contrast, seemed more likely to affiliate with a good friends' opponent.

### Effect of bystander affiliation on the type of second post-conflict interaction

**Figure 2:**
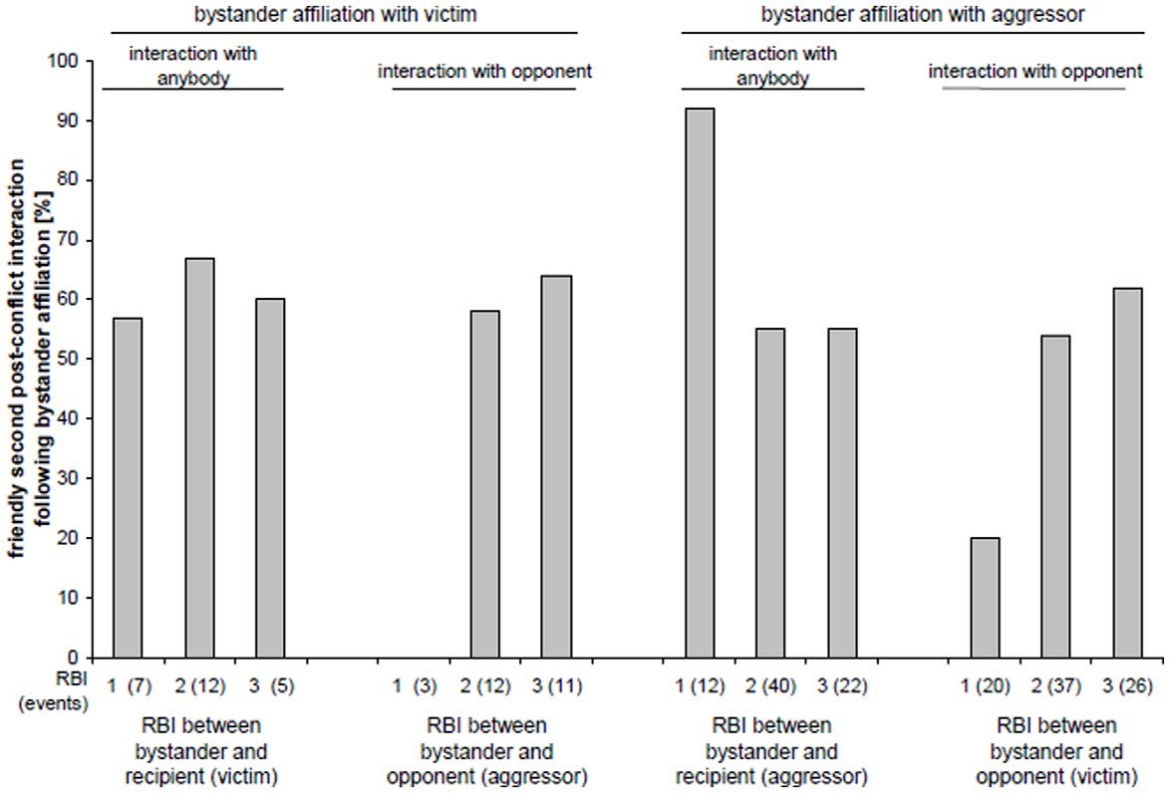
Percentage of friendly interactions during the second post-conflict interaction by the recipient of affiliation, following bystander affiliation with the victim (left) or with the aggressor (right). This is dependant on the RBI of the bystander with either the recipient of affiliation (left) or the recipient's opponent (right).

We checked for the first interaction made by the affiliation recipient following bystander affiliation. We scored whether this second post-conflict interaction was friendly or non-friendly. Likewise, we determined the first interaction amongst the former opponents following bystander affiliation. Figure 2 shows the percentage of friendly second post-conflict interactions that occurred following bystander affiliation. The interactions are grouped depending on the RBI of the bystander with both opponents and to whom the bystander had offered affiliation. We ran Generalized Linear Models (GLZ), in order to investigate whether the bystander's sex, identity or RBI with the recipient of affiliation or the opponent had any predictive power on whether or not the subsequent interaction was friendly. The results are summarized in Table 4. We did not find any predictive effect for bystander affiliation with the victim, which may be an issue of statistical power. In contrast, we found two effects after bystander affiliation had been offered to the aggressor. On one hand, opponents were significantly more likely to engage in a friendly interaction after the victim's friend had affiliated with the aggressor (Wald = 8.62, DF = 2, P = 0.013). On the other hand, the aggressor showed a tendency to engage in a friendly interaction after receiving affiliation by a non-friend (Wald = 4.62, DF = 2, P = 0.099).

**Table 4:** Results of the Generalized Linear Models (GLZ) investigating the effect of bystander's ID, sex and RBI with the two opponents on whether or not successive interactions are friendly.

Predictor	Wald	DF	P
(1) Bystander affiliation with victim			
(a) Dependent variable: successive interaction of victim			
Bystander ID	9.69	9	0.288
RBI bystander with recipient (victim)	3.35	2	0.187
Bystander sex X RBI bystander with recipient (victim)	*a*		
(b) Dependent variable: successive interaction of opponents			
Bystander ID	3.15	9	0.958
RBI bystander with opponent (aggressor)	2.89	2	0.236
Bystander sex X RBI bystander with opponent (aggressor)	*a*		
(2) Bystander affiliation with aggressor			
(a) Dependent variable: successive interaction of aggressor			
Bystander ID	19.43	13	0.110
RBI bystander with recipient (aggressor)	4.62	2	0.099
Bystander Sex X RBI bystander with recipient (aggressor)	0.36	1	0.550
(b) Dependent variable: successive interaction of opponents			
Bystander ID	12.81	13	0.462
RBI bystander with opponent (victim)	8.62	2	0.013
Bystander sex X RBI bystander with opponent (victim)	2.397	2	0.302

*a*. unable to compute due to small sample size.

X interaction between two predictors.

### Effect of bystander affiliation on the opponents' tolerance levels

In order to test whether or not tolerance amongst opponents was back to baseline levels we compared affiliation recipient's baseline time interval between consecutive affiliative interactions with the opponents' latency to have an affiliative interaction again after the bystander affiliation. We calculated the relative latency for each event, dividing the observed latency of the opponents' interaction by the corresponding baseline interval. For each RBI we separately calculated the 5% confidence limits using bootstrap sampling from the set of events. If the CI_L_≤1, then the observed latency was indifferent from baseline. If the CI_L_>1, then the observed latency was longer than baseline with a P<0.05.

**Figure 3:**
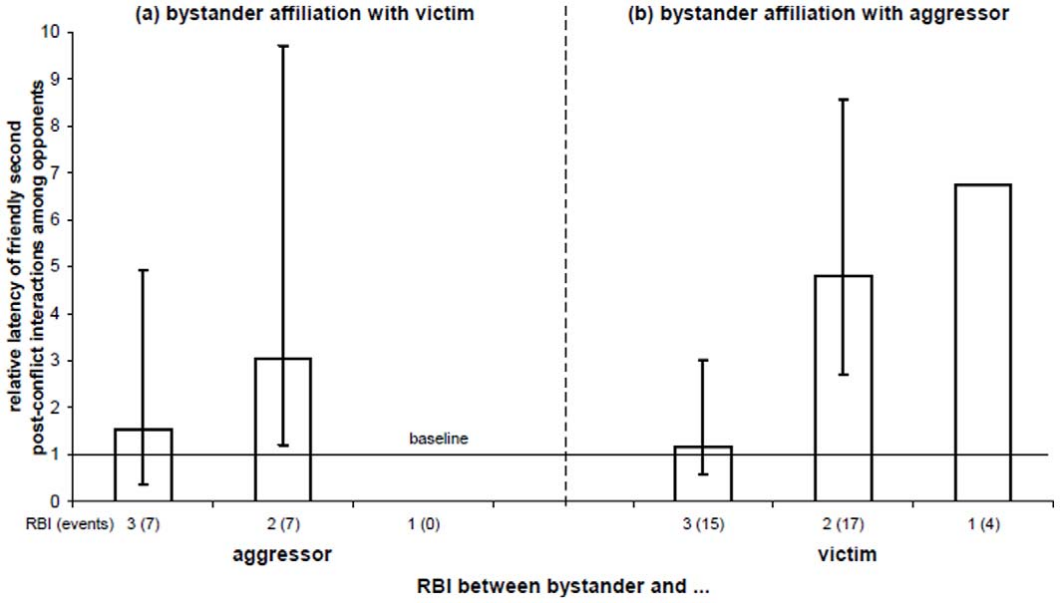
Latency of friendly second post-conflict interaction among the opponents following bystander affiliation. The data are divided in categories depending on the bystander's RBI with the recipient's opponent (RBI: 1 = no, 2 = weak, 3 = good friendship). The latency is presented in relation to the average inter-interaction time between individuals' friendly interactions (relative latency >1 indicates that post-affiliation interaction has a longer than average latency). Error lines represent the 90% confidence interval (CI) of the distribution calculated with bootstrap sampling. In case the CI excludes the value 1, opponents are significantly (p = 0.05) less tolerant with each other than under normal conditions.

First, we analyzed the bystander affiliation with the victim during the second post-conflict interaction. We found the latency between consecutive friendly interactions in first and second post-conflict interactions amongst the opponents was back to undisturbed tolerance levels only when the bystander was a good friend of the aggressor, here the recipient's opponent (Fig. 3; RBI = 3: mean relative latency of interaction  = 1.52, CI_L_  = 0.365, CI_H_  = 4.913). In cases where the bystander was only a weak friend of the aggressor (RBI = 2) the latency to the second post-conflict interaction between the opponents, when it was friendly, was significantly above baseline (Fig. 3).

We then looked at bystander affiliation with the aggressor. Tolerance between former opponents was restored to normal levels in one condition only: when the bystander was a good friend of the victim, here the recipient's opponent (Fig. 3; RBI = 3: mean relative latency of interaction  = 1.156, CI_L_  = 0.565, CI_H_  = 3.009). For both other categories (RBI = 2 and RBI = 1) the latency was significantly above baseline (Fig. 3).

## Discussion

The results fulfilled all the predictions for only one of the three hypotheses tested: the relationship repair hypothesis. Half of the predictions were met for the consolation hypothesis and no predictions were met for the self-protection hypothesis.

We found strong support for the relationship-repair hypothesis. Our data showed that both female and male bystanders were more likely to offer affiliation to the opponents of their friends. For female bystanders this occurred when affiliating with the victims of aggression and for male bystanders this occurred when affiliating with the aggressors. Former opponents were more likely to exchange a friendly interaction after a good friend of the victim had affiliated with the aggressor. These results support the relationship-repair function of bystander affiliation. In addition, the tolerance levels between former opponents were set to baseline again only when the bystander was a good friend of the opponent. This indicates that bystander affiliation in chimpanzees can substitute for direct reconciliation. All dyads in our data set (see Table 1) apart from one (RIC – NIN) did not show close kin relationships [26]. Taï chimpanzees' bystander affiliation, therefore, can function as friend-mediated reconciliation. This is similar to the kin-mediated reconciliation in savannah baboons [20] and indicates that the recipient of the bystander affiliation is aware of the quality of the relationship between the bystander and the former opponent.

Moderate support was provided for the consolation hypothesis. Both female and male bystanders also were more likely to affiliate directly with their friends. For female bystanders this occurred when affiliating with aggressors and for male bystanders this occurred when affiliating with victims. Recipients of affiliation, however, did not increase their likelihood to exchange friendly interactions following bystander affiliation. This suggests that they were not ‘calmed’ more when interacting with their good friends compared with others. Nonetheless, bystander affiliation with bonding partners has been observed in other chimpanzee and bird studies (*Pan troglodytes* [12], [27]; *Corvus frugilegus* [28]). It seems likely, that some Taï chimpanzees on some occasions were conducting bystander affiliation with a consolation outcome.

In contrast, we found no support for the self-protection function of bystander affiliation. It seems that bystanders have either good friendships with the opponents of the affiliation recipient, or they show a preference for affiliating with their good friends. We did not find that low levels of friendship explained the pattern of bystander affiliation. Although enemy relationships sometimes cause chimpanzees to act, for example when supporting the enemy of an enemy [18], socio-negative relationships don't seem to drive their bystander affiliation.

Although our data met all the predictions for only the relationship-repair hypothesis, we cannot conclude that the other two functions of bystander affiliation are absent in Taï chimpanzees. A recent study on captive chimpanzees also found that bystanders usually affiliated with their own friends, supporting the consolation function [29]. Fraser and colleagues [15] argued that each species might use several functions of bystander affiliation, but under different circumstances. It seems very likely that Taï chimpanzees apply several different functions using bystander affiliation. A larger sample size together with an experimental approach is possibly necessary to uncover all the functions and circumstances. Currently, however, we can conclude that the majority of bystander affiliations in Taï chimpanzees function to repair the relationship between former opponents. This result demonstrates the usefulness of testing the three competing hypotheses with a single data set. It highlights also the importance of examining triadic as well as dyadic relationships between the bystander and the two opponents.

Bystander affiliation in chimpanzees is usually offered when opponents are unlikely to reconcile [12], when opponents' relationship is of low benefit [22] and further aggression is more likely [22]. In these cases, opponents are likely to fission, in the absence of bystander affiliation, considering that un-reconciled conflicts leave opponents with disturbed tolerance levels [11]. It may pay bystanders to offer affiliation to the enemy of their friend in order to keep the friend who is a cooperation and grooming partner available within the party. Chimpanzees have a fission-fusion social structure, whereby ever-changing subgroups can join and separate within the home range. Individuals without group protection seem much more vulnerable to predation, especially in areas with high predation pressure (for example chimpanzees in Taï [30], [31] or baboons in Moremi [32]). They also face increased risk of lethal attacks from neighboring conspecific groups [33]. Chimpanzees are known to collaborate during group defense against neighbor attacks and the group with the most chimpanzees present is usually the victor, gaining access to preferred food sources [33], [34]. In order to enable future association or collaboration, reconciling ones' friend with the former opponent may be a crucial but self-benefiting act. This could be the reason why bystander affiliation has mainly been detected in highly social and potentially collaborative animals (e.g. *Pan troglodytes* [12], [22]; *Pan paniscus* [35]; *Gorilla beringei* [36]; *Macaca arctoides* [16]; *Papio hamadryas* [20]; *Canis familiaris* [37]; but see: *Corvus frugilegus* [28]). Thus, both reconciliation and bystander affiliation are likely to be key co-adaptations of chimpanzees' conflict management and, indeed, their sociality.

There is increasing evidence that forming and maintaining close bonds with a few other conspecifics increases the survival rates of offspring [3]–[6], [38]–[40] and increases longevity [41]. The mechanisms of how close bonds affect survival rates of offspring are still to be determined. Several studies show that individuals that maintain close bonds are better able to manage stress than those who do not, both in short and longer term analyses [42]. More effective stress management may be one mechanism to achieve higher survival rates. Different and elaborate strategies to manage the disruptive effects of aggression on group cohesion, such as direct reconciliation and reconciliation on behalf of a friend, may be other mechanisms.

## Materials and Methods

### Study site and data collection

Data were collected between October 1996 and April 1999 in the Taï chimpanzee study area, in the Taï National Park, Côte d'Ivoire, West Africa, 5°52 N and 7°22 W [26]. Since all features to test the predictions are represented in the original study on conflict management [11], [22], [43], the data set is totally suited for this additional study. In October 1996, the observed community consisted of four males (three adults, one late adolescent), 14 adult females (11 adults, three adolescents) and 13 juveniles and infants. Demography changed during the observation period. Five individuals disappeared or died (one adult male, two adolescent females, two juveniles) and six infants were born.

R.M.W. collected behavioral data on activities, social interactions and vocalizations during all-day focal animal sampling of a focal chimpanzee. On consecutive days we observed different focal animals, observing females once and males twice per month. There was, however, some variability in observation frequency due to the fission-fusion character of chimpanzee communities, death and habituation level. The result was 80 all-day follows of males (two males with 31 days each and two males with 9 days each) and 123 all-day follows of females (average 12.3 days, from max 15 to min 10 days per female). Further details in Wittig and Boesch [11], [22], [43].

### Conflict and bystander affiliation

A conflict was defined as an aggressive dyadic interaction between aggressor and victim, started by the aggressor initiating an aggressive action against the victim and ending with either submission, flight or non-aggressive behavior, which was not directly followed by further aggression. Bystander affiliation was defined as an affiliative interaction offered by the bystander (an individual not involved in the fight) to one of the opponents (called the affiliation recipient) after the conflict. Such episodes were only included in the analysis when they were the first interaction the focal chimpanzee had participated in since the conflict. It should be noted that this approach differs from some other studies, where post-conflict interactions are defined as occurring within a certain number of minutes after the conflict, irrespective of whether it is the first interaction post-conflict or not [9], [12], [44], [45]. We considered only the first post-conflict interaction as dependent on the preceding conflict, because the second post-conflict interaction, as shown here, was at least partly dependent on the first post-conflict interaction and not only on the conflict. Aggressive interactions consisted of threats (e.g. barks, arm wave), non-contact aggression (e.g. directed displays) and contact aggression (e.g. hits, bites), while affiliative interactions consisted of intimate body contact that required recipient acceptance (e.g. kiss, embrace, genital touch, grooming).

During the observation period R.M.W. observed a total of 876 conflicts between adults (following results from [22]). Bystander affiliation was the first post-conflict interaction in 164 cases, compared to 188 cases of reconciliation. Dependent offspring in chimpanzees have ranks and relationships dependent on their mother [46]. After eliminating all cases of bystander affiliation where the bystander was a sub-adult, juvenile or infant, a total of N = 109 remained for the analysis. In 26 cases the bystander offered affiliation to the victim of the aggression and in 83 events to the aggressor. It should be noted that the nature of our data collection (focal animal sampling) heavily underestimates the natural frequency of bystander affiliation. Since we were following only one of the opponents, the focal animal, we most likely missed 50% of the cases of when bystander affiliation actually occurred. Therefore, an estimate for the actual natural frequency of bystander affiliation is more likely to be around 35–40% of all conflicts.

Although bystander affiliation is a triadic interaction for some of the predictions (see Figure 1), we summarized data depending on the identity of the bystander due to two reasons: (1) we were interested in the impact of the bystander's relationship on the behavior of the opponents, and (2) to ensure that data points are independent. All adult chimpanzees were represented in the bystander data set (N = 15).

### Relationship benefit index (RBI)

Friendships, or close bonds, among social animals are measured in different ways, using various combinations of allo-grooming, association patterns or other socio-positive behaviours. Most of these measures are inter-correlated [21]. For Taï chimpanzees the Relationship Benefit Index has been statistically determined to be the best measure of the strength of a dyad's relationship [21]. The Relationship Benefit Index takes into account both agonistic support and food sharing behaviors. Both agonistic support and food sharing entail clear benefits for the receiver of the behavior and are routinely offered by an individual to only a few select others. Agonistic support is the aggressive intervention of a bystander in a conflict on behalf of one opponent against another. Receiving support can change the power balance and allowing a subordinate opponent to access the disputed resource afterwards [47], [48]. Food sharing is either an active process, by handing over a piece of food to another, or a passive process, by allowing someone to feed on high quality, monopolizable food that is in another's possession, such as meat, nuts and insects [47]. For simplicity we use the term ‘friendship’ for this relatively rare type of affiliative relationship. Pairs that supported each other and shared food were defined as ‘good friends’. Chimpanzees that shared food but did not support each other or vice versa were defined as ‘weak friends’, and dyads committed to neither were scored as ‘no friends’. The study community consisted of 105 dyads (4 males and 11 females). Nineteen were scored as being ‘good friends’, 48 were qualified as ‘weak friends’ and 38 dyads as ‘non-friends’ (Table 1). These values give the expected distribution if bystanders intervene in conflicts by chance without considering the value of their relationship to either opponent. The average RBI of an individual is calculated as the mean RBI of all dyads for this particular individual. The relationship, measured using the relationship benefit index, has been shown to influence chimpanzees' decision-making process on whether or not to act aggressively towards an opponent [43], to determine chimpanzees' reconciliatory frequency and the likelihood of bystander affiliation occurring [22], [43].

### Statistical Approach

In order to test whether two sample distributions are different we calculated the difference for each pair of samples and ran a bootstrap procedure with a 1000 repetitions. The purpose of this procedure was to calculate the 5% confidence limit for the overlapping area of the two samples. Since two samples can overlap only on one side we calculated the 90% confidence interval over the sample's average to reach a confidence limit of 5% at the side of overlap. We tested each data set of differences for normal distribution using the exact one sample Kolmogorov-Smirnov test from SPSS 17.0. All data sets of differences were classified as normal distributed by the test showing P-values between 0.988 and 0.582. Therefore we used bootstrap procedures using the mean (bootstrapped t approach [49]) for samples with N≥6. Samples of N<6 weren't statistically analyzed, but we present the sample using the median and the range of the distribution. If ‘zero’ falls within the confidence interval, the two tested samples would be indifferent. If the CI excludes ‘zero’ the two samples are different at a level of α = 0.05.

We conducted Generalized Linear Model (GLZ) analysis (Type III model effect with a maximum likelihood estimate for parameters in SPSS 17.0) to investigate how bystander affiliations affected the second post-conflict interaction. Two different dependent variables were extracted from the original data: (a) whether or not the second post-conflict interaction of the recipient was affiliative (we excluded data points with no further interactions from the recipient during the same day), and (b) whether or not the second post-conflict interaction among former opponents was affiliative (no interaction among the former opponents was scored as avoiding behaviour and therefore counted as non-friendly). Data set (a) was used to test the predicted outcome of the consolation hypothesis. Data ser (b) was used to test the predicted outcome of the relationship-repair hypothesis. Because no predictions for the self-protection hypothesis during the first post-conflict interaction were fulfilled, we did not test predictions for this hypothesis in the second post-conflict interaction. Three predictor variables were entered in the GLZs: (1) the bystander's ID, to control for individual variation, (2) the bystander's sex in an interaction with the bystander's RBI, since sex differences in the use of bystander affiliation were apparent, and (3) the bystander's RBI with either the affiliation recipient (to test for the consolation hypothesis) or the recipient's opponent (to test for the relationship-repair hypothesis). Model effects are presented in Table 4.

### Baseline for rate of friendly interactions of individuals

For each individual we calculated the average interval between two successive friendly interactions. The affiliation recipient's average interval was taken as the baseline level. We compared the baseline level with the latency of the affiliation recipient's second post conflict interaction, when it was affiliative with the former opponent. We divided the latencies of second post-conflict (affiliative) interactions by the baseline intervals. A relative latency ≤1 indicates that the latency is equal to the baseline level, while a relative latency >1 indicates that opponents needed longer than baseline to engage in friendly interactions again. We then took all relative latencies of one distribution and calculated the 90% confidence interval of the samples using bootstrap sampling with 1000 repetitions [50]. When the confidence interval excluded the value 1, we concluded that the latency of post-affiliative friendly interactions was different from baseline with an α = 0.05. Otherwise we concluded that post-affiliation friendly interactions occurred at baseline levels.

## References

[1] Krebs JR, Davis NB (1984). Behavioral ecology: an evolutionary approach (2nd edn)..

[2] Krause J, Ruxton G (2002). Living in groups..

[3] Silk JB, Alberts SC, Altman J (2003). Social bonds of female baboons enhance infant survival.. Science.

[4] Silk JB, Beehner JC, Bergman TJ, Crockford C, Engh AL (2009). The benefits of social capital: close social bonds among female baboons enhance offspring survival.. Proc R Soc B.

[5] Cameron EZ, Setsaas TH, Linklater WL (2009). Social bonds among unrelated females increase reproductive success in feral horses.. Proc Natl Acad Sci U S A.

[6] Lewis S, Roberts G, Harris MP, Prigmore C, Wanless S (2007). Fitness increases with partner and neighbour allopreening.. Biol Let.

[7] de Waal FBM (2000). Primates – A natural heritage of conflict resolution.. Science.

[8] Aureli F, van Schaik CP (1991). Post-conflict behaviour in long-tailed macaques (Macaca fascicularis): Coping with uncertainty.. Ethology.

[9] Fraser ON, Stahl D, Aureli F (2010). The function and determinants of reconciliation in *Pan troglodytes*.. Int J Primatol.

[10] Cords M (1992). Post-conflict reunions and reconciliation in long-tailed macaques.. Anim Behav.

[11] Wittig RM, Boesch C (2005). How to repair relationships – Reconciliation in wild chimpanzees Ethology..

[12] Fraser ON, Stahl D, Aureli F (2008). Stress reduction through consolation in chimpanzees.. Proc Natl Acad Sci U S A.

[13] de Waal FBM, van Roosmalen A (1979). Reconciliation and consolation among chimpanzees.. Behav Ecol Sociobiol.

[14] de Waal FM, Aureli F, Russon AE, Bard KA, Taylor Parker S (1996). Consolation, reconciliation and a possible cognitive difference between macaques and chimpanzees.. Reaching into thoughts: The minds of great apes.

[15] Fraser ON, Koski SE, Wittig RM, Aureli F (2009). Why are bystanders friendly to recipients of aggression?. Commun Integr Biol.

[16] Call J, Aureli F, de Waal FBM (2002). Postconflict third party affiliation in stumptailed macaques.. Anim Behav.

[17] Koski SE, Sterck EH (2009). Post-conflict third-party affiliation in chimpanzees: What's in it for the third party?. Am J Primatol.

[18] de Waal FBM, van Hooff JARAM (1981). Side-directed communication and agonistic interactions in chimpanzees.. Behaviour.

[19] Judge P (1991). Dyadic and triadic reconciliation in pigtailed macaques (*Macaca nemestrina*).. Am J Primatol.

[20] Wittig RM, Crockford C, Wikberg E, Seyfarth RM, Cheney DL (2007). Kin-mediated reconciliation substitutes for direct reconciliation in female baboons.. Proc R Soc B.

[21] Wittig RM, Lonsdorf EV, Ross SR, Matsuzawa T (2010). Function and cognitive underpinnings of post-conflict affiliation in wild chimpanzees.. The mind of the chimpanzee: Ecological and experimental perspectives.

[22] Wittig RM, Boesch C (2003). The choice of post-conflict interactions in wild chimpanzees (*Pan troglodytes*).. Behaviour.

[23] Aureli F, Schaffner C (2002). Relationship assessment through emotional mediation.. Behaviour.

[24] Crockford C, Wittig RM, Whiten PL, Seyfarth RM, Cheney DL (2008). Social stressors and coping mechanisms in wild female baboons (*Papio hamadryas ursinus*).. Horm Behav.

[25] Wittig RM, Crockford C, Lehmann J, Whiten PL, Seyfarth RM (2008). Focused grooming networks and stress alleviation in wild female baboons.. Horm Behav.

[26] Boesch C, Boesch-Achermann H (2000). The chimpanzees of the Taï Forest..

[27] Kutsukake N, Castles DL (2004). Reconciliation and post-conflict third-party affiliation among wild chimpanzees in Mahale Mountains, Tanzania.. Primates.

[28] Seed AM, Clayton NS, Emery NJ (2007). Postconflict third-party affiliation in rooks, *Corvus frugilegus*.. Curr Biol.

[29] Romero T, Castellanos MA, de Waal FBM (2010). Consolation as possible expression of sympathetic concern among chimpanzees.. Proc Natl Acad Sci U S A.

[30] Boesch C (1991). The effect of leopard predation on grouping patterns in forest chimpanzees.. Behaviour.

[31] Hill K, Boesch C, Goodall J, Pusey A, Williams J (2001). Mortality rates among wild chimpanzees.. J Hum Evol.

[32] Cheney DL, Seyfarth RM, Fischer J, Beehner J, Bergman T (2004). Factors affecting reproduction and mortality among baboons in the Okavango Delta, Botswana.. Int J Primatol.

[33] Boesch C, Crockford C, Herbinger I, Wittig R, Moebius Y (2008). Intergroup conflicts among chimpanzees in Taï National Park: Lethal violence and female perspective.. Am J Primatol.

[34] Mitani JC, Watts DP, Amsler SJ (2010). Lethal intergroup aggression leads to territorial expansion in wild chimpanzees.. Curr Biol.

[35] Palagi E, Paoli T, Borgonini S (2004). Reconciliation and consolation in captive bonobos (*Pan paniscus*).. Am J Primatol.

[36] Watts D (1995). Post-conflict social events in wild mountain gorillas. II. Redirection, side direction and consolation.. Ethology.

[37] Cools AKA, van Hout AJM, Nelissen MHJ (2008). Canine reconciliation and third-party-initiated postconflict affiliation: do peacemaking social mechanisms in dogs rival those of higher primates?. Ethology.

[38] Silk JB, Beehner JC, Bergman TJ, Crockford C, Engh AL (2009). The benefits of social capital: close social bonds among female baboons enhance offspring survival.. Proc R Soc B.

[39] Weidt A, Hofmann SE, Koenig B (2008). Not only mate choice matters: fitness consequences of social partner choice in female house mice.. Anim Behav.

[40] Hawkes K (2004). The grandmother effect.. Nature.

[41] Silk JB, Beehner JC, Bergman TJ, Crockford C, Engh AL (2010). Strong and consistent social bonds enhance the longevity of female baboons.. Curr Biol.

[42] Cheney DL, Seyfarth, RM (2009). Stress and coping mechanisms in female primates.. Adv Stu Behav.

[43] Wittig RM, Boesch C (2003). ‘Decision-making’ in conflicts of wild chimpanzees: An extension of the Relational Model.. Behav Ecol Sociobiol.

[44] Preuschoft S, Wang X, Aureli F, de Waal FBM (2002). Reconciliation in captive chimpanzees: A re-evaluation with controlled methods.. Int J Primatol.

[45] Koski SE, Sterck EHM (2007). Triadic postconflict affiliation in captive chimpanzees: Does consolation console?. Anim Behav.

[46] Goodall J (1986). The chimpanzees of Gombe: Patterns of behavior..

[47] Wittig RM, Boesch C (2003). Food competition and linear dominance hierarchy among female chimpanzees of the Taï National Park.. Int J Primatol.

[48] Nishida T, Hosaka K, McGrew WC, Marchant L, Nishida T (1996). Coalition strategies among adult male chimpanzees of Mahale Mountains, Tanzania.. Great ape societies.

[49] Efron B, Tibshirani RJ (1993). Introduction to the bootstrap..

[50] DiCiccio TJ, Efron B (1996). Bootstrap confidence intervals.. Stat Sci.

